# ELIMINATE: a PCR record-based macroelimination project for systematic recall of HCV-RNA-positive persons in Austria

**DOI:** 10.1007/s00508-023-02275-4

**Published:** 2023-09-29

**Authors:** Caroline Schwarz, David Bauer, Livia Dorn, Mathias Jachs, Lukas Hartl, David Chromy, Lukas Weseslindtner, Nikolaus Pfisterer, Barbara Hennlich, Annika Stückler, Robert Strassl, Astrid Voill-Glaninger, Wolfgang Hübl, Martin Willheim, Karin Köhrer, Sonja Jansen-Skoupy, Sabine Tomez, Walter Krugluger, Christian Madl, Michael Schwarz, Lorenz Balcar, Georg Semmler, Leonard Brinkmann, Lukas Burghart, Lukas Antonitsch, Gerhard Weidinger, Florian Riedl, Hermann Laferl, Vesselina Kurteva, Marianna Traugott, Julian Hind, Christoph Wenisch, Abdelrahman Aburaia, Christian Sebesta, Daniela Schmid, Sonja Rothweiler, Jelena Remetic, Michael Gschwantler, Andreas Maieron, Thomas Reiberger

**Affiliations:** 1 Division of Gastroenterology and Hepatology, Department of Medicine III, Medical University of Vienna, Waehringer Guertel 18–20, 1090 Vienna, Austria; 2 Vienna HIV & Liver Study Group, Medical University of Vienna, Vienna, Austria; 3 Department of Internal Medicine IV, Klinik Ottakring, Vienna, Austria; 4 Internal Medicine 2, Gastroenterology and Hepatology and Rheumatology, Karl Landsteiner University of Health Sciences, University Hospital of St. Pölten, St. Pölten, Austria; 5 Department of Dermatology, Medical University of Vienna, Vienna, Austria; 6 Center for Virology, Medical University of Vienna, Vienna, Austria; 7 Department of Internal Medicine IV, Klinik Landstraße, Vienna, Austria; 8 Clinical Institute for Laboratory Medicine, Medical University of Vienna, Vienna, Austria; 9 Central Laboratory and Blood Bank, Klinik Landstraße, Vienna, Austria; 10 Central Laboratory, Klinik Ottakring, Vienna, Austria; 11 Clinical Institute of Laboratory Medicine, University Clinic St. Pölten, St. Pölten, Austria; 12 Institute of Medical-Chemical and Molecularbiological Laboratory Diagnostics with Blood Depot, Landesklinikum Wiener Neustadt, Wiener Neustadt, Austria; 13 Institute of Laboratory Diagnostics, Klinik Favoriten, Vienna, Austria; 14 Institute of Laboratory Medicine with Blood Depot, Klinik Donaustadt, Vienna, Austria; 15 Institute of Laboratory Medicine and Blood Depot, Klinik Floridsdorf, Vienna, Austria; 16 Sigmund Freud University, Vienna, Austria; 17 Department of Internal Medicine, Gastroenterology and Hepatology, Landesklinikum Wiener Neustadt, Wiener Neustadt, Austria; 18 Department of Internal Medicine IV, Klinik Favoriten, Vienna, Austria; 19 Department of Internal Medicine and Gastroenterology, Klinik Floridsdorf, Vienna, Austria; 20 Department of Internal Medicine II, Klinik Donaustadt, Vienna, Austria; 21 Österreichische Agentur für Gesundheit und Ernährungssicherheit GmbH (AGES), Vienna, Austria; 22 Gilead Sciences, Vienna, Austria

**Keywords:** Hepatitis C virus, Chronic hepatitis C, Elimination, Macroelimination, Direct-acting antivirals

## Abstract

**Background and aims:**

Micro-elimination projects targeted to specific hepatitis C virus (HCV) risk populations have been successful. Systematic identification of persons with HCV viremia, regardless of risk group, based on already available laboratory records may represent an effective macroelimination approach to achieve global HCV elimination.

**Methods:**

Persons with a last positive HCV-RNA PCR result between 2008–2020 in the reference virology laboratories in eastern Austria were identified. First, (i) we described their demographic characteristics, (ii) we systematically recalled persons to the respective centers and (iii) started antiviral treatment if HCV-RNA viremia was confirmed, and (iv) recorded sustained virologic response (SVR). This interim report includes the preliminary results from 8 participating centers.

**Results:**

During the study period 22,682 persons underwent HCV-RNA PCR testing, 11,216 (49.4%) were positive at any point in time, and 6006 (26.5%) showed detectable HCV-RNA at the last PCR test, suggesting ongoing HCV viremia.

At the time of this interim report, 2546/6006 HCV-RNA PCR(+) persons were evaluated: 443/2546 (17.4%) had died, 852/2546 (33.5%) had invalid contact data, and 547/2546 (21.5%) had achieved SVR between data retrieval and recall. Contact could be established in 236/704 (33.5%) of the remaining target population with 97/236 (41.1%) presenting at the clinic for treatment evaluation. Ultimately, 71/236 (30.1%) started antiviral treatment and SVR was documented in 47/71 (66.2%).

**Conclusion:**

This ELIMINATE project based on systematic assessment of HCV-RNA PCR-records, identified 6006 persons with potential persisting HCV viremia. Invalid contact data and missed visits for treatment evaluation were the main barriers towards HCV elimination within this project. Importantly, many subjects with HCV viremia lost to follow-up were successfully linked to care and started antiviral treatment.

## Introduction

Despite the declaration of global hepatitis C virus (HCV) elimination by 2030 as a WHO public health goal and the availability of directly acting antivirals (DAA) facilitating sustained virologic response (SVR) rates of > 95%, HCV remains a major global health burden [1] with 56.8 million people infected worldwide [2–6].

The current prevalence of HCV in Austria is estimated at 82,746 (95%-Confidence interval: 67.9–100.4) persons [7]. Numerous HCV microelimination projects that specifically target high-risk populations such as people who inject drugs (PWID), prison inmates, homeless people [8–11], and men who have sex with men (MSM) [9, 12] have been initiated in Austria over the past years [12–16]:

The PWID on opioid agonist therapy (OAT) were offered point-of-care HCV screening integrated into their OAT prescription process [13, 14, 17]. Subsequently, they received “directly observed therapy” for HCV: DAA were administered alongside PWIDs’ established OAT under the supervision of pharmacists/healthcare workers. Through this ongoing project, drug adherence increased and so far 600 PWID in Vienna successfully achieved sustained virological response (SVR) with DAAs (SVR rate 99.0%) [17].

Since 2020, HCV screening is offered to all homeless people frequenting the multidisciplinary low threshold institution “Neunerhaus” in Vienna [18]. DAA treatment is offered to all HCV viremic patients and is provided for those without health insurance [16]. To facilitate drug adherence, housing is provided during DAA treatment. So far, 400 homeless people were screened and HCV viremia was detected in 10.8% [16].

To remove information and treatment barriers, a physician-based hotline operated by specialists at the viral hepatitis clinic of the Medical University of Vienna offers a low-barrier tool for communication and linkage to care [15, 19]. In 2019, 79 antiviral therapies with an SVR4 rate of 98.0% were initiated via this “HepC Phone” [15].

While these microelimination projects target specific at-risk populations in Austria, the estimated remaining national prevalence of HCV suggests a considerable number of persons with HCV viremia remain without antiviral treatment [8]. Various previous studies showed that HCV-infected persons are lost throughout all stages of the HCV cascade of care [20] and have not been sufficiently linked to care [21]. As DAA-based HCV therapies are covered by the universal Austrian health insurance, the major remaining barriers are identification of HCV viremic persons and linkage to treatment.

Screening and treatment utilization must be further improved in Austria, as elimination of HCV does not seem achievable without macroelimination projects, i.e., approaches targeting an unselected population irrespective of risk factors for HCV infection. We present an interim report of an ongoing macro-elimination project in eastern Austria (“ELIMINation AusTria East”, ELIMINATE), targeting a “submerged” population unaware of their HCV infection or those diagnosed with HCV, but not yet sufficiently linked to care.

## Patients and methods

The aim of this study is the epidemiological description of the remaining persons with chronic HCV infection in eastern Austria and the systematic recall of these persons to offer HCV therapy with DAA. The project consists of two parts: (i) systematic analysis of HCV testing results from participating centers in Lower Austria, Vienna, and Burgenland and (ii) the evaluation for and initiation of DAA treatment in persons showing HCV-RNA viremia through an active recall initiative.

### Part 1: patient characteristics of identified persons with potential persistent HCV-RNA viremia in eastern Austria

**Fig. 1:**
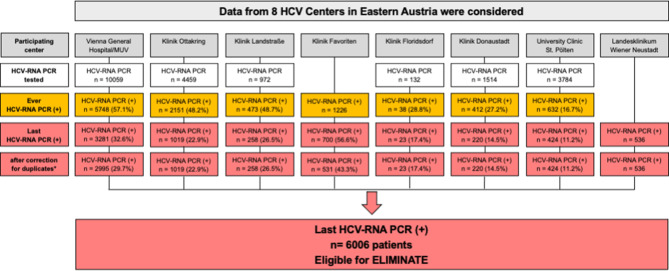
Flowchart of the screened population. HCV-RNA PCR results by participating center (acquired 2008–2020). Preliminary data as of December 2022. For Klinik Favoriten (Vienna), the total number of HCV-RNA PCR tests performed during the observation period was not available, therefore calculations were based on the number of persons with “ever HCV-RNA PCR (+)” (i.e., persons who were HCV-RNR-PCR positive at any point in time). For Landesklinikum Wiener Neustadt (Lower Austria), only the number of persons with “last HCV-RNA PCR (+)” was available. *HCV* hepatitis C virus; *RNA* ribonucleic acid; *PCR* polymerase chain reaction; *MUV* Medical University of Vienna, *asterisk* persons who received HCV-RNA PCR testing at more than one center were assigned to the center where the last chronological test had been performed for further analyses

In Austria, only certified HCV treatment centers are authorized to prescribe DAA therapy. Certified HCV institutions as well as all hospitals that regularly perform HCV tests in Lower Austria, Vienna and Burgenland were invited to participate in this project. Currently, 8 centers *(*Fig. 1*)* have provided data for this study, and 12 additional centers have declared their future cooperation.

Data were acquired through systematic retrieval of laboratory data by each center. We assessed all available anti-HCV serologies and all HCV-RNA PCR tests performed between January 2008 and the date of data retrieval (which naturally varies between centers, ranging from December 2018 Vienna General Hospital to April 2020 Klinik Ottakring) including the date of testing and the test result (anti-HCV serology: positive/negative; HCV-RNA PCR: positive/negative/quantitative). The numbers of anti-HCV serologies and HCV-RNA PCR tests were assessed overall and for each center, and the percentages of positive results were calculated. Chronic hepatitis C was defined as HCV-RNA PCR positive (+) with or without an available anti-HCV serology. SVR was defined as HCV-RNA PCR negative (−) after previous anti-HCV(+) or HCV-RNA PCR(+). To put our data into perspective, estimations of the overall HCV prevalence in Austria were performed based on the Global Burden of Disease Study 2017 using modelling via the Coalition for Global Hepatitis Elimination [22]. The proportions were assigned to federal states by the proportion of population according to the 2021 data from Statistik Austria [23].

All persons whose last chronological HCV-RNA PCR result was positive (considering all centers where data were available at the time of preliminary analysis) were included in the active recall part of the project (“screened population”).

### Part 2: recall initiative for evaluation and initiation of antiviral treatment in persons with confirmed HCV-RNA viremia

Individual databases were generated for each participating center, containing the data from all persons whose last HCV-RNA PCR result had been positive at the respective center.

Based on these individual databases, persons were contacted by telephone or mail and were invited for HCV assessment at their treatment center by a local HCV physician. For medical institutions that are not licensed as HCV treatment centers but where HCV viremia was detected through diagnostic evaluation, the respective patients were recalled by a collaborating HCV treatment center.

Among all persons evaluated so far, those who had died or achieved SVR between data retrieval and data assessment as well as all persons with invalid contact data were excluded from the “screened population”. The remaining persons who last showed a positive HCV-RNA PCR (“target population”) were invited to the respective HCV treatment center and were offered:

1. Laboratory evaluation including platelet count, liver biochemistry tests, virologic parameters (HCV, HIV, HBV).
2. Liver stiffness measurement using elastography (Fibroscan®, Echosens, France).
3. Counselling regarding HCV transmission and treatment options.

Persons in whom HCV viremia was detectable by the assessment were offered treatment with direct-acting antivirals (DAAs). The choice of DAA was made according to individual comorbidities, comedications, previous HCV treatments, prescriber and person preferences. Routine surveillance data under DAA treatment were recorded, including dynamics in HCV-RNA PCR and elastography. SVR was defined as negative HCV-RNA PCR after the end of DAA treatment. Numbers and percentages of persons called, reached, clinically assessed, treated, and cured (SVR) were evaluated overall and for each center.

Patient HCV status on recall (persisting HCV viremia/SVR) and contact status (reached vs. not reached) were recorded. Furthermore, the suspected route of HCV transmission was assessed.

### Statistical methods

Data bank management and descriptive statistics were performed using Microsoft Excel 16.63.1 (2022 Microsoft, Redmond, WA, USA). The R language and environment for statistical computing and graphics (version 4.0.0+) including the dplyr-library were utilized for person selection and statistics. To identify persons tested and or treated at more than one center, so as to avoid double counts and double recalls, we compared centers databases based on an unique identifier created from the first name, the last name and the date of birth and assigned persons present in more than one database to the center where the last positive test had been performed. Figures were generated using Graph Pad Prism 9 (GraphPad Software, La Jolla, CA, USA) and Microsoft PowerPoint 16.63.1 (2022 Microsoft). Categorical variables were reported as the number of persons with/without (proportion of persons with) the characteristic of interest. Case numbers, frequencies, and percentages were calculated. Continuous variables were reported as median and interquartile range (IQR).

## Results

### Part 1: results of the systematic record-based assessment of virology data

**Fig. 2:**
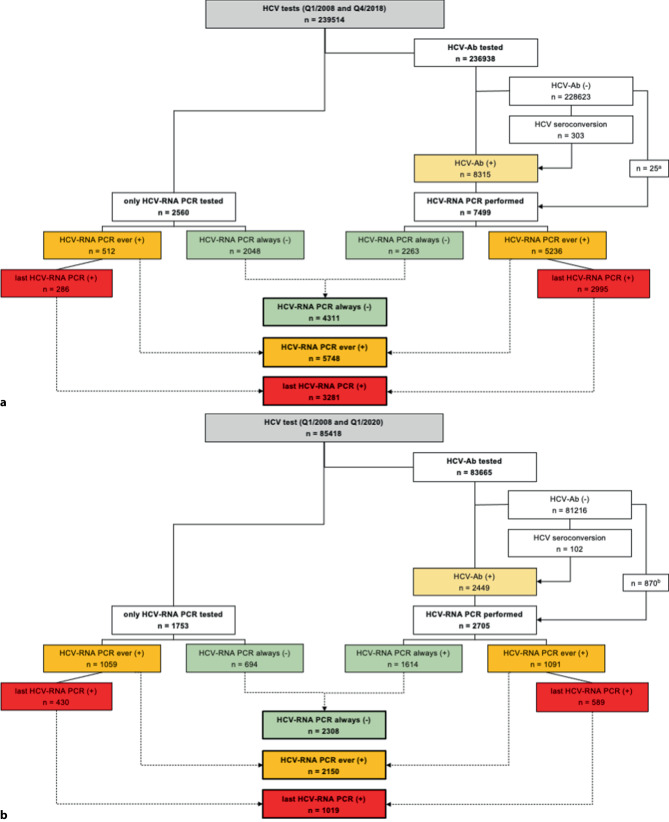
HCV seroprevalence and HCV viremia in eastern Austria. Flow chart of persons evaluated by HCV serology and/or HCV-RNA PCR at the Vienna General Hospital of the Medical University of Vienna, the Center for Virology of the Medical University of Vienna and Klinik Ottakring (Vienna).** a** HCV testing at the Vienna General Hospital between January 2008 and December 2018, ^a^ including 4/25 (16.0%) with HCV-RNA(+).** b** HCV testing at Klinik Ottakring (Vienna) between November 2008 and April 2020, ^b^ including 25/870 (2.9%) with HCV-RNA(+). *HCV* hepatitis C virus; *HCV-Ab* hepatitis C virus antibodies; *RNA* ribonucleic acid; *PCR* polymerase chain reaction

The eight participating centers provided data for this preliminary analysis. For two centers more detailed analyses based on both anti-HCV serology and HCV-RNA PCR data were available (Fig. 2a, b). These centers also provided data on the distribution of HCV genotypes (GT).

#### HCV seroprevalence and HCV viremia (Fig. 2a, b, 3a, b)

**Fig. 3:**
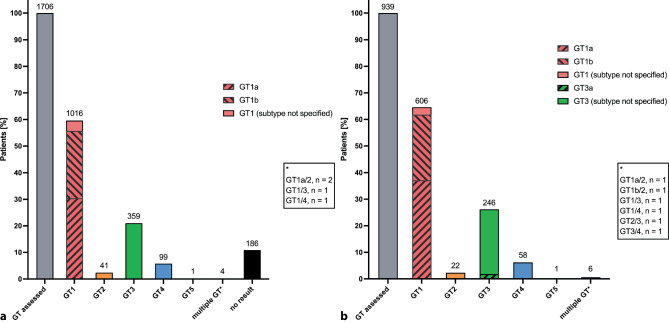
HCV genotype assessment. **a** Distribution of HCV genotypes among the “screened population” at the Vienna General Hospital.** b** Distribution of HCV genotypes among the “screened population” at Klinik Ottakring. Numbers of persons tested for the respective HCV genotype. HCV genotype evaluation was available for a total of *n* = 2645 individuals. *GT* genotype

Between January 2008 and December 2018 a total of 239,514 persons received an HCV test at the Vienna General Hospital (Fig. 2a) and 236,938 (98.9%) received an HCV serology test, showing a seroprevalence of seropositivity of 3.5% (8315). Additionally, 303 (0.1%) persons experienced HCV seroconversion during the observational period, resulting in a total of 8618 (3.6%) anti-HCV (+) persons. Of the 8643 (8618 anti-HCV(+) and 25 anti-HCV(−) persons), 7499 (87.0%) were followed-up with an HCV-RNA PCR: 5236 (69.8%) of them ever showed an HCV-RNA PCR(+) and in 2995 (39.9%) persons the last available HCV-RNA PCR result was positive. Furthermore, 2560 of 239,514 (1.1%) received an HCV-RNA PCR without prior HCV serology: 512/2560 (20.0%) ever showed HCV-RNA PCR(+) and in 286 (11.2%) persons the last HCV-RNA PCR was positive. Therefore, the total HCV-RNA(+) population for the Vienna General Hospital of the Medical University of Vienna and the Center for Virology of the Medical University of Vienna was estimated at 3281 (referred to as “screened population” in Part 2 of the study) of 10,059 (32.6%) HCV-RNA PCR tested individuals. Among the 1706/3281 (52.0%) HCV viremic individuals who underwent HCV-GT assessment, GT1 (59.6%) was most frequently found (Fig. 3a).

At Klinik Ottakring, 85,418 persons received an HCV test between November 2008 and April 2020, 83,665 (97.9%) were tested via HCV serology, among which 2449 (2.9%) presented with anti-HCV(+) (Fig. 2b) and 102 (0.1%) experienced HCV seroconversion during the observational period, resulting in a total of 2551 of 83,665 (3.0%) anti-HCV (+) subjects. Out of 3421 (2551 anti-HCV(+) and 870 anti-HCV(−) persons) 2705 (79.1%) were further evaluated by HCV-RNA PCR: 1091 (40.3%) had ever shown HCV-RNA PCR(+) and 589 (21.7%) had HCV viremia at the last PCR evaluation. In addition, 1753 of 85,418 (2.1%) individuals were tested via HCV-RNA PCR without prior serological assessment for anti-HCV: 1059 of 1753 (60.4%) of them ever presented with HCV viremia and in 430 (24.5%) the last available HCV-RNA PCR was positive. The total HCV-RNA(+) population for Klinik Ottakring, was 1019 (referred to as “screened population” in Part 2 of the study) of 4458 (22.9%) HCV-RNA PCR-tested subjects. Among the 939/2150 (43.7%) HCV viremic individuals in whom HCV-GT assessment was performed, GT1 (64.5%) was most frequently detected (Fig. 3b).

#### Population of patients with potentially persisting HCV viremia (Fig. 1)

At the 8 centers included in this interim analysis, a total of 22,682 persons received at least 1 HCV-RNA PCR test between 2008 and 2020 (Fig. 1). Of these persons 11,216 (49.4%) had at least 1 HCV-RNA PCR(+) result during the observation period and in 6229 (27.5%) cases, the last available HCV-RNA PCR showed a positive result. For Klinik Favoriten, the total number of HCV-RNA PCR tests performed during the observation period was not available, therefore calculations were based on the number of persons who ever showed a positive HCV-RNA PCR. For Landesklinikum Wiener Neustadt, only the number of persons whose last HCV-RNA PCR showed a positive result was available. In 217/6229 (3.5%) persons who last showed a positive HCV-RNA PCR, the corresponding individuals had received testing at more than 1 center and, hence, were assigned to the center where the last chronological HCV-RNA PCR had been performed. After correction for these “duplicates”, 6006 persons with potentially persisting HCV viremia comprised the “screened population” for our HCV macroelimination project.

### Part 2: recall initiative for evaluation and initiation of antiviral treatment in persons with confirmed HCV-RNA viremia

**Fig. 4:**
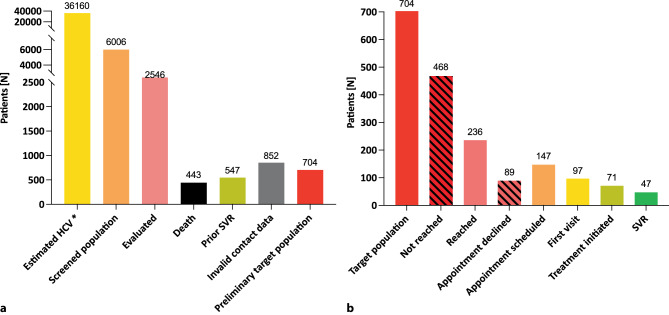
Preliminary work-up of the “screened population”. Evaluation of the “screened population” and assessment of the “target population” in the context of the estimated number of people living with HCV and the current progress of the ELIMINATE project at the time of preliminary data analysis (preliminary data as of December 2022).** a** Determination of the preliminary “target population”. *Asterisk* Institute for Health Metrics and Evaluation (IHME) via https://www.globalhep.org/country-progress/austria. **b** Cascade of care based on the preliminary “target population”. *HCV* hepatitis C virus; *SVR* sustained virologic response; *LTFU* lost to follow-up

All 6006 persons who last showed a positive HCV-RNA PCR were included in the active recall part of the project (“screened population”; 16.6% of the estimated 36,160 people living with HCV in eastern Austria [7], Fig. 4a). At the time of preliminary data analysis, 2546/6006 (42.4%) persons had been evaluated: 443 (17.4%) subjects had died and 547 (21.5%) had achieved SVR since data retrieval. In 852 (33.5%) individuals recall failed due to invalid contact data. Ultimately, the “target population” (last HCV-RNA PCR(+) verified, contact data available) consisted of 704 (27.7%) persons:

The characteristics of this *n* = 704 “target population” are summarized in Table 1. Briefly, 59.5% were male, the median age was 42 (IQR: 19) years. Importantly, 86/591 (14.6%) who had a fibrosis evaluation (transient elastography and/or FIB‑4 score [24] and/or APRI score [25] available) showed advanced F3/F4 fibrosis. HCV-GTs were predominantly GT‑1 (*n* = 145, 60.9) and GT‑3 (*n* = 65, 27.3%) among the 238/704 (33.8%) who underwent GT assessment. Unfortunately, route of HCV transmission and previous HCV treatment were not comprehensively available, however, intravenous drug use (204/256, 79.7%) was the most common transmission route and the majority (359/416, 86.3%) of patients were HCV treatment naive.

**Table 1:** Baseline characteristics of the study population at last follow-up prior to recall

	Overall
**Patients, *n*** **(%)**	704 (100)
**Sex, *n*** **(%)**	
Male	419 (59.5)
Female	285 (40.5)
**Age (years), median (IQR)**	42.0 (19)
**HCV-RNA viral load (IU/mL), median (IQR)**	605,000 (1,721,000)
**Qualitative HCV-RNA PCR positive, *n*** **(%)**	33 (4.7)
**HCV genotype, *n*** **(%)**^**a**^	
1	145^bc^ (60.9)
2	10^b^ (4.2)
3	65 (27.3)
4	19^c^ (8.0)
6	1 (0.4)
**Liver stiffness by TE**^**d**^	
*Median LSM (kPa, IQR)*	6.8 (3.6)
*Fibrosis stage*^*e*^	
F0/1	95 (50.5)
F2	41 (21.8)
F3	15 (8.0)
F4	37 (19.7)
**APRI score**^**f**^	
Median (IQR)	0.4 (0.4)
APRI score > 1.5	57 (9.9)
**FIB‑4 score**^**g**^	
Median (IQR)	1.0 (1.0)
FIB‑4 score > 3.25	77 (13.4)
**Advanced fibrosis, *n*** **(%)**^**h**^	86 (12.2)
**Route of HCV transmission, *n*** **(%)**^**i**^	
IDU	204 (79.7)
Nasal drug use	2 (0.8)
Blood products	31 (12.1)
MSM	3 (1.2)
Heterosexual transmission	9 (3.5)
Tattoo or piercing abroad	3 (1.2)
Needle injury	4 (1.6)
**Previous HCV treatment, *n*** **(%)**^**j**^	
*DAA*	
Non-response	5 (1.2)
LTFU	26 (6.3)
*PEG-IFN*	
Nonresponse	19 (4.6)
LTFU	7 (1.7)
*Treatment-naive*	359 (86.3)

*HCV* hepatitis C virus; *RNA* ribonucleic acid; *PCR* polymerase chain reaction; *TE* transient elastography; *LSM* liver stiffness measurement; *APRI score* aspartate-aminotransferase to platelet ratio index; *FIB‑4 score* fibrosis‑4 score; *IDU* intravenous drug use; *MSM* men who have sex with men; *DAA* direct-acting antivirals; *LTFU* lost to follow-up; *PEG-IFN* pegylated interferon

^a^ available in *n* = 238 (33.8%)

^b^ including *n* = 1 patient with HCV genotype 1 and 2 coinfection.

^c^ including *n* = 1 patient with HCV genotype 1 and 4 coinfection.

^d^ available in *n* = 188 (26.7%)

^e^ the following cut-offs were used for fibrosis stage classification: < 7.1 for F0/F1 (absent/mild fibrosis), ≥ 7.1 kPa and < 9.5 kPa for F2 (significant fibrosis), ≥ 9.5 kPa and < 12.5 kPa for F3 (severe fibrosis) and ≥ 12.5 kPa for F4 (cirrhosis).

^f^ *n* = 574 (81.5%) available data, upper limit of normal for AST: 35 IU/L (females) and 50 IU/L (males)

^g^ *n* = 573 (81.4%) available data

^h^ defined by either LSM ≥ 9.6 kPa, or APRI > 1.5, or FIB4 > 3.25; *n* = 113 (16.1%) missing data

^i^ *n* = 256 (36.4%) available data

^j^ *n* = 416 (59.1%) available data

Among the “target population”, 468 (66.5%) could not be reached using the available contact data (Fig. 4b). While recall was successful in 236 (33.5%) persons and 89 of 236 (37.7%) declined the offered clinical treatment evaluation. The remaining 147 (20.9%) persons received an appointment for treatment evaluation and 97 of 147 (66.0%) have already attended their first visit at the time of data analysis: 71 of 97 (73.2%) started treatment with DAA, in 47 (66.2%; accounting for 6.7% of the preliminary “target population”) of which SVR could be documented, while 24 (33.8%) were lost to follow-up after the end of treatment.

## Discussion

This regional HCV macroelimination project focuses on a heterogeneous group of persons consisting mostly of people unaware of the HCV infection who were lost to follow-up despite established diagnosis of hepatitis C [21].

Based on estimations according to the latest available IHME data, we expect about 36,160 people to live with HCV infection in the region covered by our project [7], only 16.6% of which are included in our “screened population”; however, we expect to increase this percentage as more centers continue to provide data. Consequently, the number of persons among the “evaluated population” and the “target population” will increase as data processing and active recall proceed in the currently and prospectively participating centers.

A considerable percentage among the “evaluated population” died before recall (17.4%), likely explained by testing in patients hospitalized for severe extrahepatic and hepatic conditions, but potentially also by to the detrimental effects of HCV infection on survival. A main barrier for our approach was insufficient contact data in 33.5% of the “evaluated population”. While mortality analyses will be performed in the final report of the project after data completion, first estimations revealed a high rate of death at advanced age and a relevant percentage of PWID who died in association with adverse drug events [9, 26]. This distribution may change with further data integration as currently most evaluated data came from centers in Vienna, a city in which intravenous drug use (IDU) may account for more HCV infections than in rural areas of eastern Austria [27].

Notably, 21.5% of the “evaluated population” achieved SVR between data retrieval and recall, demonstrating the effectiveness of ongoing local HCV elimination strategies [2, 12–14, 28]. Of note, our definition of chronic hepatitis C as any positive HCV-RNA RCR technically also includes acute hepatitis C cases, therefore we cannot rule out that some of the individuals who cleared HCV viremia between data retrieval and recall may have had acute hepatitis C. As a matter of fact, the constellation of anti-HCV(−) and HCV-RNA(+) found among 29 individuals at the Vienna General Hospital and at Klinik Ottakring could be representative of the serological window of acute HCV infection rather than real occult HCV infections, as the rate of up to 16.0% among those with anti-HCV(−) seems very high for occult HCV infections [29]; however, the median time between data retrieval and recall exceeded 6 months in all participating centers, hence we do not expect a relevant number of acute hepatitis C cases among our “evaluated population”. Meanwhile, 37.7% of the persons reached declined treatment evaluation. The description of the reasons for declining linkage to care will be subject to the final analyses; however, preliminary estimations revealed a high rate of advanced age and/or immobility as well as other acute health problems that had to be prioritized. As the project was mainly performed during the ongoing COVID-19 pandemic, associated restrictions and people’s fear of infection also played a major role in the decline of appointments [30]. Furthermore, a relevant number of persons reported subjectively compromising alcohol dependence, which had to be addressed first to enable DAA adherence [13, 14]. Importantly, all persons who declined treatment evaluation received counselling on HCV transmission, liver disease evaluation/management as well as on treatment options and were invited to make a new appointment at any time.

Among the 71 persons who started DAA treatment, 47 (66.2%) achieved SVR, while 24 (33.8%) remain within the posttreatment observational period. As modern DAA treatment leads to SVR rates > 95% across all risk groups [13, 14], it seems likely that the overwhelming majority of these persons will also achieve SVR.

Consistent with global and national estimates, GT1 (60.9%) and GT3 (27.3%) were detected most frequently among the evaluated population, according to available data [31]. Similarly, GT5 and GT6 were not relevant among the evaluated population; however, GT4 (8.0%) infections appear to have increased slightly compared to the previous literature, which may be attributable to recent immigration dynamics in Austria [31].

To our knowledge, this is the first Austrian HCV macroelimination project based on systematic recall regardless of risk behavior [32]. Retrospectively expanding this approach over more than 10 years facilitates a great coverage of individuals at risk, while the multicenter setting strengthens collaborations between healthcare institutions.

However, the project also has some limitations: First, participation is based on individual consent of each institution and not compulsory for all diagnostic/treatment institutions in the covered region. Therefore, we will naturally miss HCV tests performed at institutions not participating in the project. Second, not all parameters are comprehensively available, making some comparisons/analyses impossible. Third, name cross-matching across centers may not be entirely robust towards different spelling and variants of names. Fourth, the preliminary results acquired so far may not be representative of the entire “screened population” as identification and recall efforts are still ongoing.

In summary, systematic identification of persons with potential persisting HCV viremia represents a successful macroelimination approach and supports the achievement of the WHO elimination goals for HCV [21]. This project will facilitate linkage to care in a relevant number of HCV-infected persons who might otherwise never have undergone HCV treatment or liver disease evaluation. Furthermore, it will create regional epidemiological data to inform future targeted elimination strategies. The systematic approach based on available HCV-RNA PCR records used in this macroelimination project in eastern Austria seems widely applicable to other countries, thereby supporting global HCV elimination.
